# Fluid Extract Kidney Leaf Comp.

**Published:** 1877-08-20

**Authors:** Wm. A. Greene

**Affiliations:** Macon, Ga.


### FLUID EXTRACT KIDNEY LEAF COMP.

by Wm. A. Greene, Macon, Ga.

I feel that it is incumbent on me to make the following report:

"Bright's disease, and its treatment, are still among the *vexatal questiones* of Pathology and Therapeutics. Dr. Bright, of London, first pointed out in 1827, the frequent connexion which exists between dropsy, and what was afterwards termed granular degeneration of the kidney, and presence of albumen in the urine, as an indication of the latter lesion. But Dr. G. Johnson, of King's College detected the real nature of this most prevalent and fatal disease, and more modern pathologists have made yet further discoveries of its *morbid anatomy*. It is a slow, insidious disease, beginning generally much further back than the patient is aware of. It would be impossible, within the limits of this paper, to follow the minutiæ of the pathology, morbid anatomy, constitutional symptoms, state of the urine, consequences, causes and treatment of this perplexing disease. I propose here, with what has been said, only a brief reference to its treatment, with a "new remedy" which accidentally fell in to my hands, or rather, which I *accidentally used*. It is called by the manufacturers, "*Fluid Extract Kidney Leaf Comp.*"—and made according to the following formula, consequently no *secret* remedy or nostrum.

R. Fluid extract kidney—leaf....oz. xv.
Testa mellusca.......... ...oz. 1¼.
Nitrate potassa...... .......grs. 100.

Mix.

Dose, for an adult, one table-spoonful from three to six times in twenty-four hours, in a half tumbler of warm milk, being certain to keep bowels open once or twice a day, with some saline cathartic.

The circumstances causing me first to employ this remedy are as follows:

I had been for many months treating a gentleman affected with Bright's disease. His disease had become chronic and pronounced by several intelligent physicians to be *Bright's disease*. He had a pallid and pasty complexion, weakness and drowsiness; indigestion and frequent, almost constant nausea—trying to vomit first thing in the morning after rising; palpitation of the heart; unusual anæmia, and awakened frequently during the night to pass water. Anasarca had already appeared, with some ascites. Urine decidedly albuminous, with occasional red particles of blood. Specific gravity, as usual, very low, from 1012 to 1014, and under the microscope contained fatty epitheleum scales. Urine was becoming scanty, and I anticipated speedy dissolution from uræmic poisoning. He had been in the hands of the best physicians, and I knew had taken all the usual remedies. His case had been given up as beyond all cure, as is generally too often the case, when arriving at the stage in which I found this poor man. My rule in practice is to never look or appear surprised at anything, and never give up a patient or quit dosing them. I first gave him freely of liquor ammoniæ acetatis, as so highly extolled at Guy's Hospital when this affection was first discovered, and since then been most carefully studied. It has been found by Dr. Addison that by acting on the skin, and thus relieving the congested condition of the kidneys, this was the best of all remedies, used in England, diuretics, at the same time, being avoided. I also put him under the mild influence of protiodide hyd., with ext. conium. Failing to benefit him, or even arrest the progress of the disease, I next gave him large doses of lime water, theoretically, from its having the property of dissolving protein. Under this treatment the albumen sensibly diminished, but the slight appearance of blood in the urine necessitated the disuse of this treatment. Not knowing what to recommend the above-named medicine came to my attention, and being determined to use anything that gave the remotest promise of relief I concluded to give it an honest trial. I procured as quickly as possible two bottles of the medicine and directed him to take one tablespoonful every four hours in half tumblerful of warm milk, and if he improved to extend the interval to six hours and report again in two weeks. At the same time I gave him a bottle of beef, iron and wine with lactopeptine. In the mean time I corresponded with Messrs Reed and Carnrick for further information concerning the medicine, and also with intelligent physicians of good professional position, who had used the remedy successfully in the same disease. He returned (living some distance from Macon) after taking the contents of both bottles, to my great surprise, vastly improved in all respects. I furnished more medicine (same kind) with the addition of a bottle of granular effervescent bicarb. potass. to keep his bowels open. This treatment was begun in May 1877, and at this writing, being about three months, the improvement is sufficient to encourage the hope of complete restoration to health.

A second case, just entering the second stage of the disease, is taking the same treatment with even more benefit.

A third case of ovarian dropsy in a lady, two years ago in robust health, in whom the cyst had been twice emptied at an interval of eight months, urine decidedly albuminous, specific gravity 1018, is under similar treatment with decided benefit.

I have prescribed it in other cases of kidney and urinary trouble with satisfactory results. But for the fact of this being one of our standard and first-class manufacturing pharmaceutical houses, dealing entirely with physicians, I should not have noticed this preparation, even after my attention had been called to it by practitioners of good standing, who had tested its virtues. I have certainly seen enough of its therapeutic effects on this class of diseases to warrant the publication of my observation and experience, especially since its successful treatment has baffled the best skill, and become an opprobrium in medicine. While chemical investigations are most valuable and should not be overlooked in determining the value of remedial agents, yet testimony from facts and cases, after all, is the best and most reliable evidence.

I earnestly ask those having such cases to give this remedy a trial. It is being prescribed in various sections of the country by the best physicians, and with almost universally satisfactory results. I have taken considerable trouble to investigate the medicine, and see no more impropriety in physicians using it than the sulphate of cinchonidia, or cincho quinine.

